# LFVB-BioSLAM: A Bionic SLAM System with a Light-Weight LiDAR Front End and a Bio-Inspired Visual Back End

**DOI:** 10.3390/biomimetics8050410

**Published:** 2023-09-05

**Authors:** Ruilan Gao, Zeyu Wan, Sitong Guo, Changjian Jiang, Yu Zhang

**Affiliations:** 1 State Key Laboratory of Industrial Control Technology, College of Control Science and Engineering, Zhejiang University, Hangzhou 310027, China; gaoruilan@zju.edu.cn (R.G.); zeyuwan@zju.edu.cn (Z.W.); guositong@zju.edu.cn (S.G.); changjianjiang@zju.edu.cn (C.J.); 2 Key Laboratory of Collaborative Sensing and Autonomous Unmanned Systems of Zhejiang Province, Hangzhou 310027, China

**Keywords:** simultaneous localization and mapping (SLAM), bionic robotics, loop closure detection, path integration

## Abstract

Simultaneous localization and mapping (SLAM) is one of the crucial techniques applied in autonomous robot navigation. The majority of present popular SLAM algorithms are built within probabilistic optimization frameworks, achieving high accuracy performance at the expense of high power consumption and latency. In contrast to robots, animals are born with the capability to efficiently and robustly navigate in nature, and bionic SLAM algorithms have received increasing attention recently. Current bionic SLAM algorithms, including RatSLAM, with relatively low accuracy and robustness, tend to fail in certain challenging environments. In order to design a bionic SLAM system with a novel framework and relatively high practicality, and to facilitate the development of bionic SLAM research, in this paper we present LFVB-BioSLAM, a bionic SLAM system with a light-weight LiDAR-based front end and a bio-inspired vision-based back end. We adopt a range flow-based LiDAR odometry as the front end of the SLAM system, providing the odometry estimation for the back end, and we propose a biologically-inspired back end processing algorithm based on the monocular RGB camera, performing loop closure detection and path integration. Our method is verified through real-world experiments, and the results show that LFVB-BioSLAM outperforms RatSLAM, a vision-based bionic SLAM algorithm, and RF2O, a laser-based horizontal planar odometry algorithm, in terms of accuracy and robustness.

## 1. Introduction

The last 3 decades have witnessed remarkable progress in the research area of autonomous robot navigation. Simultaneous localization and mapping (SLAM), as one of the fundamental techniques utilized by robots to perform navigation tasks, refers to the process of constructing the map of the unknown environment that the robot is exploring and estimating the pose of the robot within it simultaneously [1,2,3]. As illustrated in the survey [4], the architecture of a SLAM system can be divided into two major parts: the front end and the back end. The front end receives sensor data and converts it into models suitable for robot state estimation, while the back end conducts inference on the abstracted data generated by the front end.

Currently, most popular SLAM algorithms typically conduct the back-end inference under probabilistic filtering or nonlinear optimization framework [5]. The sensor modalities they utilize to obtain environmental information include LiDARs [6], radars [7], cameras (e.g., standard RGB cameras [8], RGB-D cameras [9], infrared cameras [10] and event-based cameras [11]), and inertial measurement units (IMUs). Moreover, multi-sensor fusion approaches have attracted growing attention.

The LiDAR and radar enhance the reliability of spatial distance information by actively transmitting and receiving signals (laser beams and radio waves), thereby improving the accuracy of the SLAM algorithm at the expense of higher power consumption. LiDAR-based SLAM algorithms mainly include LOAM [12], LIO-SAM [6], and Fast-LIO2 [13].

SLAM algorithms based on standard RGB cameras use the data association between multiple frames of images to accomplish robot ego-motion estimation and environment mapping. Representative visual SLAM (vSLAM) algorithms include ORB-SLAM [8,14,15], DSO [16], and VINS-Mono [17]. The robustness and accuracy of vSLAM are constrained by environmental lighting conditions, making it challenging to handle scenarios with motion blur and high dynamic range (HDR) lighting conditions.

The event camera, as a novel type of biologically-inspired visual sensor modality, generates asynchronous pulse event outputs only when significant illumination changes are detected at each independent pixel. Consequently, event-based SLAM algorithms, such as EVO [18], ESVO [11], and Ultimate-SLAM [19], offer advantages including low power consumption, low latency, high dynamic response speed, and resistance to motion blur [20]. However, there is still a long way to go for the community to advance the development of event-based SLAM.

As described by Cadena et al. in their survey [4], the development of SLAM has gone through three main periods: the classical age (1986–2004), the algorithmic-analysis age (2004–2015), and the robust-perception age (since 2016). Despite the considerable development of SLAM research over the last 30 years, the robustness of SLAM algorithms still falls short when dealing with challenging environments, significantly limiting their applications in practice. Moreover, traditional SLAM techniques heavily rely on sufficient computing resources and power supply, further restricting their applicability.

In contrast to robots, animals possess the innate capability to navigate efficiently and robustly in natural environments by leveraging a range of sensory cues. Consequently, there has been a growing interest in developing SLAM algorithms that draw inspiration from the neural mechanisms employed by animals during navigation. By emulating the neural activities underlying animals’ navigation, these bionic SLAM algorithms strive to enhance the performance and adaptability of robotic systems in challenging real-world scenarios.

RatSLAM [21,22] is a bio-inspired SLAM algorithm that derives insights from the hippocampal model of rodents and abstracts the concept of pose cells to represent the position and orientation of a mobile robot in a 2D environment. The RatSLAM system can be deployed in real robots and has successfully accomplished autonomous localization and mapping tasks in indoor office environments [23] and outdoor suburban areas [24].

Following RatSLAM, a series of relevant studies have been completed. Inspired by the three-dimensional navigation neural representation mechanisms found in birds and bats, Yu et al. proposed a four-degree-of-freedom NeuroSLAM [25] system as an extension of RatSLAM for mapping 3D environments. In order to address RatSLAM’s sensitivity to perceptual aliasing caused by the low-dimensional sensory template matching, the LatentSLAM [26] system proposes an unsupervised representation learning method generating low-dimensional latent state descriptors and improves the robustness of the SLAM system by combining multiple sensors.

Spiking neural networks (SNNs) offer a suitable solution for feature extraction and descriptor representation for loop closure detection in SLAM. Safa et al. fused the data from an event camera and a radar, which is preprocessed by an SNN with continual spike-timing-dependent plasticity (STDP) learning, with the output used as feature descriptor encoding for loop closure detection and map correction in the SLAM architecture [27]. SNNs can also be applied in template matching and image-based place recognition. Hussaini et al. proposed a high-performance dual-layer SNN model with a novel weighted assignment scheme for visual place recognition (VPR) tasks, which can provide robust, energy-efficient, and low-latency robot localization [28].

SLAM algorithms running on neuromorphic hardware have been developed recently. Tang et al. proposed a brain-inspired SNN architecture that solves the uni-dimensional SLAM problem by introducing spike-based reference frame transformation, visual likelihood computation, and Bayesian inference [29]. Integrated to Intel’s Loihi processor, it performs well in terms of accuracy and energy-efficiency. Kreiser et al. introduced an SNN-based one-dimensional path integration architecture and implemented it on the neuromorphic hardware ROLLS [30]. Subsequently, Kreiser et al. extended this work to achieve two-dimensional path integration and map formation using an SNN architecture that comprises spiking neurons and plastic synapses, demonstrating the feasibility of the neuromorphic realization of SLAM [31].

In this paper, we propose LFVB-BioSLAM, a first-of-its-kind bionic SLAM system with a light-weight LiDAR-based front end and a bio-inspired vision-based back end. A range flow-based LiDAR odometry algorithm is adopted as the front end of the SLAM system, providing essential odometry estimation information for the back end, and a bio-inspired back-end processing algorithm based on the monocular RGB camera is proposed, performing loop closure detection and path integration. Experiments on the platform of a ground mobile robot are carried out to validate the feasibility of the proposed algorithm. The experimental results show that LFVB-BioSLAM outperforms RatSLAM [21,22], a vision-based bionic SLAM algorithm, and RF2O [32], a laser-based horizontal planar odometry algorithm, in terms of accuracy and robustness.

The main contributions of this paper are outlined below:

- We employ a lightweight range flow-based LiDAR odometry as the front end of our SLAM system, which quickly generates horizontal planar odometry output using the 3D LiDAR point cloud input.
- Our SLAM system incorporates a bio-inspired visual loop closure detection and path integration algorithm as the back end, which utilizes the odometry estimation from the front end, along with image input, to generate the robot’s pose and construct the environmental map.
- We propose a novel bionic SLAM system called LFVB-BioSLAM, which combines the aforementioned front and back end components. Through real-world experiments, we validate the feasibility of LFVB-BioSLAM and demonstrate its superior performance in terms of accuracy and robustness.

## 2. Materials and Methods

**Figure 1:**
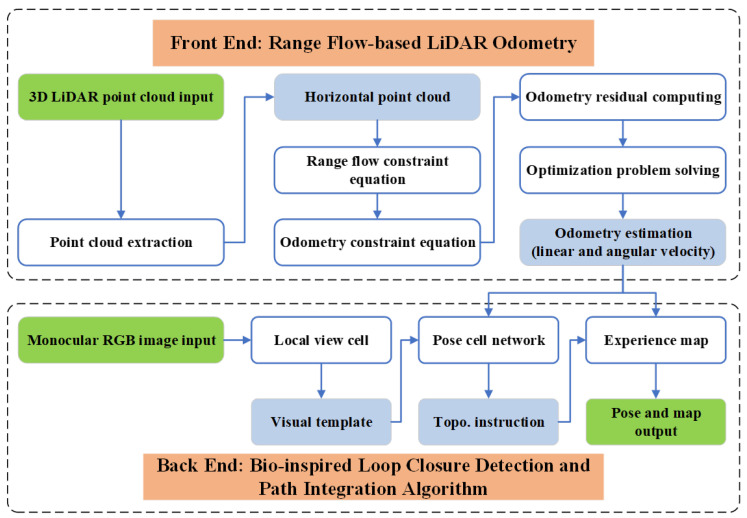
The LFVB-BioSLAM system architecture. The front-end range flow-based LiDAR odometry takes the 3D LiDAR point cloud as input, calculating the horizontal planar odometry estimation. The back-end bio-inspired visual loop closure detection and path integration algorithm takes both the monocular RGB image and the odometry estimation as input, outputting the pose of the robot and the map of the environment.

We herein describe the proposed LFVB-BioSLAM, the system architecture of which is illustrated in Figure 1, in detail as follows. Section 2.1 introduces the light-weight range flow-based LiDAR odometry algorithm, which is the front end of the LFVB-BioSLAM system. Section 2.2 describes the bio-inspired visual loop closure detection and path integration algorithm, which constitutes the back end of LFVB-BioSLAM.

### 2.1. Front End: A Light-Weight Range Flow-Based LiDAR Odometry Algorithm

This section introduces the LiDAR odometry algorithm, which generates horizontal planar odometry output, i.e., the linear and angular velocity of the robot, from the 3D LiDAR point cloud input.

First, the required horizontal single-ring point cloud is extracted from the 3D LiDAR point cloud input. Next, a range flow constraint equation is established, with the horizontal planar velocities of the robot as variables. Finally, a robust cost function is applied to minimize the geometric constraints derived from the constraint equation, thereby generating the odometry estimation output.

#### 2.1.1. Horizontal Single-Ring Point Cloud Extraction

**Figure 2:**
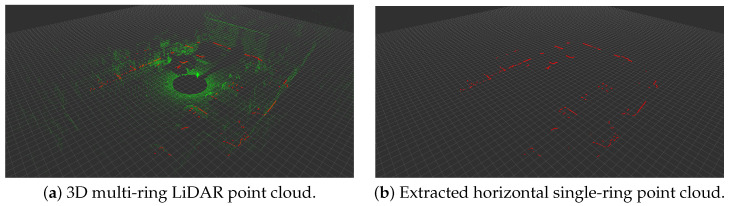
An example of horizontal single-ring point cloud extraction.

In order to use the results of the 3D LiDAR odometry (LO) algorithm as the benchmark for validating the designed algorithm, we adopt an Ouster 3D LiDAR in our sensor system. Within the input 3D multi-ring LiDAR point cloud (as shown in Figure 2a), the extraction of the required horizontal single-ring point cloud (as shown in Figure 2b) is performed, aiming to achieve a similar effect to that obtained by using a horizontal laser scanner.

Additionally, since our ground mobile robot platform equipped with the sensor system lacks corresponding planning and control algorithms and requires remote control by an operator, points generated by the robot itself and the operator are removed during the extraction process.

Let *P* denote the multi-ring LiDAR point cloud in one scan and *L* denote the horizontal single-ring point cloud. Let $P_{i}$ represent the point cloud on the *i*th ring, i.e., $P_{i}=\left\{p_{i,1},p_{i,2},\ldots ,p_{i,n_{i}}\right\}$, where $n_{i}$ is the number of points on the *i*th ring and $p_{i,j}$ denotes the *j*th point on the *i*th ring. The required horizontal single-ring point cloud, *L*, can be expressed as

(1) $$L=\overset{m}{\underset{i=1}{⋃}}L_{i}=\overset{m}{\underset{i=1}{⋃}}\left\{p_{i,j}|-h_{0}\leq h\left(p_{i,j}\right)\leq h_{0},d\left(p_{i,j}\right)\geq d_{0}\right\}$$

where *m* is the number of rings, and $L_{i}$ is horizontal single-ring point cloud extracted from the *i*th ring, i.e., the points on the *i*th ring with their heights between $-h_{0}$ and $h_{0}$ and their distances from the LiDAR no less than $d_{0}$. $h_{0}$ and $d_{0}$ are positive thresholds for height and distance, respectively.

#### 2.1.2. Range Flow Constraint Equation

The following horizontal planar LO algorithm adopted in this paper draws inspiration from RF2O [32], a fast and precise laser-based horizontal planar odometry algorithm that estimates the 2D odometry based on consecutive range scans from a laser scanner.

At time *t*, the position of a scanned point *P* in {L}, the local reference frame of the LiDAR, is determined by its polar coordinates P(r,θ), with $r\in \left(0,+\infty \right)$, and $\theta \in \left[-\frac{Fov}{2},\frac{Fov}{2}\right]$, where Fov is the field of view of the horizontal planar single-ring point cloud. The scan range of point *P* can be expressed as R(t,θ)=r.

Assuming the scan range function *R* is differentiable, we apply a first-order Taylor expansion to the scan range of point with the polar angle θ+δθ in the subsequent scan:

(2) $$R\left(t+\delta t,\theta +\delta \theta \right)=R\left(t,\theta \right)+\frac{\partial R}{\partial t}\left(t,\theta \right)\cdot \delta t+\frac{\partial R}{\partial \theta }\left(t,\theta \right)\cdot \delta \theta +o\left(\delta t,\delta \theta \right)$$

where δt denotes the time interval between two adjacent scans and δθ denotes the change in polar angle of the considered point. By ignoring the higher-order infinitesimal term o(δt,δθ) and dividing both sides of the equation by δt, the scan gradients can be related to the changes in range and polar angle within the time period [t,t+δt]:

(3) $$\frac{\delta R}{\delta t}=R_{t}+R_{\theta }\frac{\delta \theta }{\delta t}$$

where $R_{t}=\frac{\partial R}{\partial t}\left(t,\theta \right)$ and $R_{\theta }=\frac{\partial R}{\partial \theta }\left(t,\theta \right)$ represent the scan gradients for *t* and θ, respectively.

When the time interval δt between two adjacent scans is sufficiently small, the changes in scan range and polar angle within the time period [t,t+δt] divided by δt can be approximated by their corresponding derivatives, respectively, i.e., $\frac{\delta R}{\delta t}=\dot{r}$, $\frac{\delta \theta }{\delta t}=\dot{\theta }$. Thus, we have

(4) $$\dot{r}=R_{t}+R_{\theta }\dot{\theta }$$

which is the range flow constraint equation [33].

**Figure 3:**
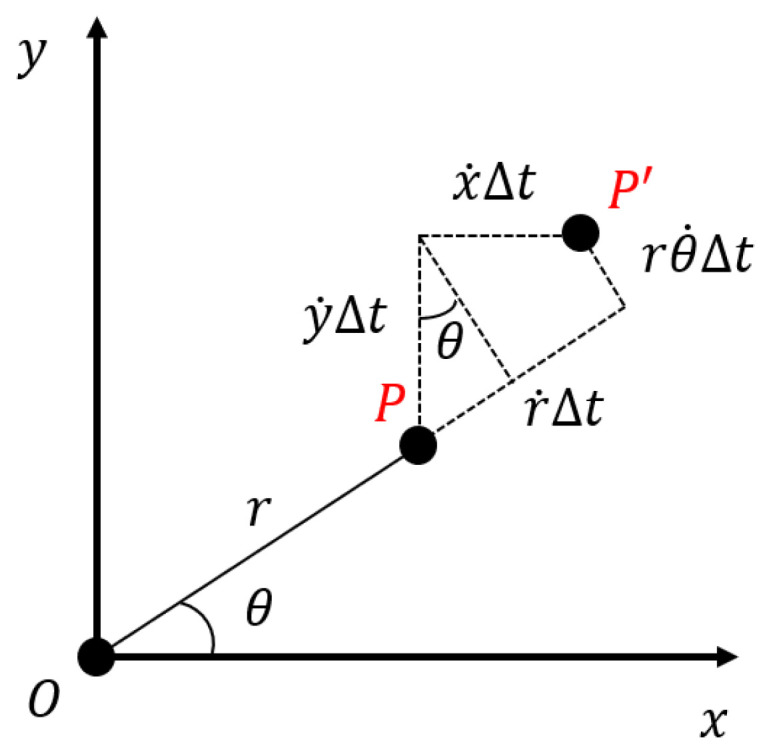
The vertical view of the local reference frame of the LiDAR. Assuming the LiDAR is stationary, the change in observation is caused by the motion from point *P* to point $P^{\prime }$ after a time interval δt.

Next, Equation (4) will be transformed into the constraint equation for the robot’s planar motion velocity (linear and angular velocity). First, the polar representation will be converted to its equal Cartesian representation. Let the Cartesian coordinates of point *P* in the local reference frame of the LiDAR be (x,y), and assuming that the LiDAR is stationary, the velocity of point *P* in the *x* and *y* directions are denoted by $\dot{x}$ and $\dot{y}$, respectively, as illustrated in Figure 3. Therefore, we have

(5) $$\left[\begin{array}{c} \dot{r}\\ \dot{\theta } \end{array}\right]=\left[\begin{array}{cc} \cos\theta & \sin\theta \\ -\frac{\sin\theta }{r} & \frac{\cos\theta }{r} \end{array}\right]\left[\begin{array}{c} \dot{x}\\ \dot{y} \end{array}\right]$$

Considering the assumption of a static and rigid environment, the change in observation is in fact caused by the ego-motion of the LiDAR (both translational and rotational motion). Let the velocity of the LiDAR in the *x* and *y* directions be denoted as $v_{x}$ and $v_{y}$, respectively, and the angular velocity be denoted as ω. Then, we have

(6) $$\left[\begin{array}{c} \dot{x}\\ \dot{y} \end{array}\right]=\left[\begin{array}{ccc} -1 & 0 & y\\ 0 & -1 & -x \end{array}\right]\left[\begin{array}{c} v_{x}\\ v_{y}\\ \omega \end{array}\right]$$

By substituting the velocity transformation (6) into the polar-Cartesian transformation (5) and substituting $\dot{r}$ and $\dot{\theta }$ into the range flow constraint Equation (4), we have

(7) $$\left(\cos\theta +\frac{R_{\theta }\sin\theta }{r}\right)\cdot v_{x}+\left(\sin\theta -\frac{R_{\theta }\cos\theta }{r}\right)\cdot v_{y}-R_{\theta }\cdot \omega +R_{t}=0$$

which is the range flow constraint equation, with the LiDAR’s planar motion velocities (linear velocity $v_{x}$, $v_{y}$, and angular velocity ω) being the variables.

#### 2.1.3. Estimation of Odometry

It is evident that in Equation (7), three sets of linearly independent constraints are theoretically sufficient to estimate the robot’s motion velocity. However, in practice, this approach is not feasible due to scan noise, linear approximation errors, and interference from moving objects in dynamic environments. These factors prevent Equation (7) from being an exact equality in typical situations. Therefore, we formulate the estimation of odometry as an optimization problem.

Let the robot’s planar motion velocity be denoted as $v={\left[v_{x},v_{y},\omega \right]}^{T}$. For each observed point in the scan and a given v to be optimized, a range-flow residual can be constructed as

(8) $$\psi \left(v\right)=\left(\cos\theta +\frac{R_{\theta }\sin\theta }{r}\right)\cdot v_{x}+\left(\sin\theta -\frac{R_{\theta }\cos\theta }{r}\right)\cdot v_{y}-R_{\theta }\cdot \omega +R_{t}$$

The optimal estimate $v^{*}$ can be obtained by minimizing the sum of robust cost functions of all range-flow residuals:

(9) $$v^{*}=\underset{v}{\arg\;\min}\sum_{i=1}^{N}F\left(\psi_{i}\left(v\right)\right)$$

with *N* denoting the number of scanned points, and the robust cost function F(ψ) defined as

(10) $$F\left(\psi \right)=\left\{\begin{array}{cc} \frac{\psi^{2}}{k^{2}}\left(1-\frac{\psi^{2}}{2k^{2}}\right), & \mathrm{if}\;|\psi |<k,\\ \frac{1}{2}, & \mathrm{if}\;|\psi |\geq k. \end{array}\right.$$

where *k* is a tunable positive parameter for decreasing the effects of outliers.

The optimization problem is then solved using the iteratively reweighted least squares (IRLS) algorithm. In each iteration, it re-computes the residuals and updates the weights until convergence is achieved.

### 2.2. Back End: A Bio-Inspired Visual Loop Closure Detection and Path Integration Algorithm

This section introduces the bio-inspired visual loop closure detection and path integration algorithm based on a monocular RGB camera, building upon the light-weight front-end odometry estimation algorithm discussed in Section 2.1.

First, the monocular RGB image is passed to the local view cell module, where the visual template corresponding to that specific scene is generated. Then, the odometry estimation and the visual template are jointly passed to the pose cell network module, which outputs topological graph instructions (creating new nodes, creating new edges, or setting nodes). Finally, the odometry estimation and the topological graph instructions are jointly passed to the experience map module, where map construction, graph relaxation, and path integration are performed, outputting the robot’s pose and the constructed environmental map.

The bio-inspired back-end processing algorithm implemented in this paper draws inspiration from RatSLAM [21], emulating the neural processes in the hippocampus of mammalian brains during navigation. It utilizes image data from a monocular RGB camera and combines it with the odometry estimation from the front-end LiDAR odometry algorithm to perform loop closure detection and path integration, outputting the robot’s pose and the constructed environmental map.

#### 2.2.1. Local View Cell

**Figure 4:**
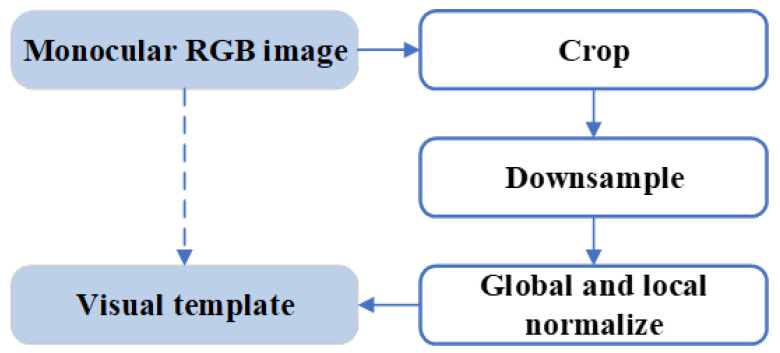
The image preprocessing procedure. First, we crop the image. By removing areas such as skies and roads, we focus on the regions of interest that are rich in environmental features, which can better repressent the visual scene of the environment. Next, we downsample the cropped image, reducing its size while preserving important visual information of the environment. Last, we perform both global and local normalization on the downsampled image to minimize the effect of the illumination variation and ensure consistency of the visual template.

Each local view cell is associated with a unique visual scene in the environment, which is intended to determine whether the current scene in the current view is a new scene or a previously seen one by comparing the input RGB image. In practice, we first preprocess the monocular RGB image, converting it into a corresponding visual template, based on which the determination of whether the current visual scene is new or previously seen is conducted. The image preprocessing procedure is illustrated in Figure 4.

**Figure 5:**
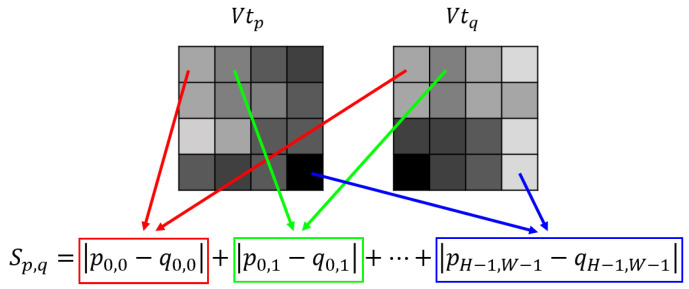
Computation of similarity between two visual templates ${Vt}_{p}$ and ${Vt}_{q}$.

After the preprocessing steps, the local view cell compares the visual template ${Vt}_{n+1}$ representing the current scene with all the historical visual templates (${Vt}_{1},{Vt}_{2},\ldots ,{Vt}_{n}$). The similarity comparison is based on the sum of absolute differences (SAD) between the current visual template and each historical visual template. For examlpe, the SAD between visual templates ${Vt}_{p}$ and ${Vt}_{q}$, denoted as $S_{p,q}$, is computed as

(11) $$S_{p,q}=\sum_{i=0}^{H-1}\sum_{j=0}^{W-1}\left|p_{i,j}-q_{i,j}\right|$$

where $p_{i,j}$ and $q_{i,j}$ are the pixel values of ${Vt}_{p}$ and ${Vt}_{q}$ respectively, with *H* and *W* being the height and width of the visual templates, respectively, which can be further illustrated in Figure 5.

If the minimum SAD among all the historical visual templates is below a predetermined threshold, then the current visual template is supposed to represent the same visual scene as the corresponding historical visual template. Otherwise, if all SAD values are above the threshold, then the current visual template is added to the historical visual templates as a representation of the current new visual scene.

#### 2.2.2. Pose Cell Network

**Figure 6:**
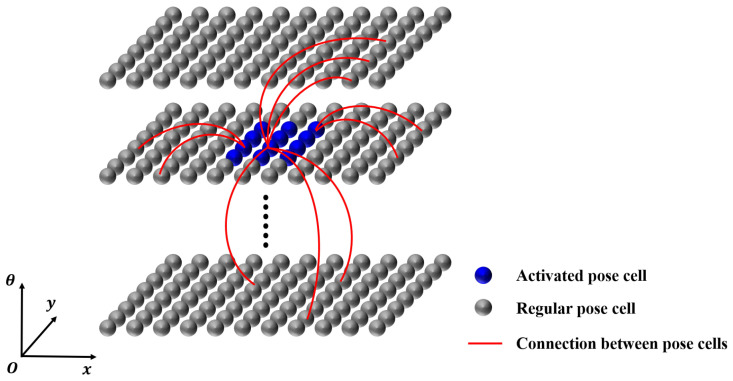
The pose cell network. Individual pose cells are interconnected with either excitatory or inhibitory connections. This network represents the three dimensions of a ground mobile robot’s pose, i.e., *x*, *y*, and θ.

The pose cell network draws inspiration from cells related to navigation (e.g., grid cells [34], place cells [35] and head direction cells [36]) found in the hippocampus of mammalian brains. It is built in the form of a continuous attractor network (CAN), as illustrated in Figure 6.

The pose cell network has artificial dynamics designed to guide its behavior. When the network reaches a stable state, a cluster of activated pose cells forms a single energy packet, as shown by the dark blue region in Figure 6. The centroid of the energy packet represents the optimal estimate of the robot’s current pose within the network. The dynamics mechanism is implemented through the local excitation and global inhibition of the continuous attractor network, achieved by weighted connections with three-dimensional Gaussian distribution excitation coefficients $\epsilon_{a,b,c}^{\mathrm{exc}}$ and inhibition coefficients $\epsilon_{a,b,c}^{\mathrm{inh}}$, respectively, which are defined as

(12) $$\epsilon_{a,b,c}^{\mathrm{exc}}=e^{-\frac{a^{2}+b^{2}}{k_{p}^{\mathrm{exc}}}}\cdot e^{-\frac{c^{2}}{k_{o}^{\mathrm{exc}}}}$$

(13) $$\epsilon_{a,b,c}^{\mathrm{inh}}=-e^{-\frac{a^{2}+b^{2}}{k_{p}^{\mathrm{inh}}}}\cdot e^{-\frac{c^{2}}{k_{o}^{\mathrm{inh}}}}$$

where $k_{p}$ and $k_{o}$ are constants related to the spatial distribution variance of the pose cell network, and the subscripts “p” and “o” represent “position” and “orientation”, respectively.

Consequently, guided by the internal dynamics of the pose cell network, the changes in pose cell energies resulting from local excitation and global inhibition mechanisms are given by

(14) $$\delta P_{x,y,\theta }^{\mathrm{exc}}=\sum_{i,j,k}\left(P_{i,j,k}\cdot \epsilon_{a,b,c}^{\mathrm{exc}}\right)$$

(15) $$\delta P_{x,y,\theta }^{\mathrm{inh}}=\sum_{i,j,k}\left(P_{i,j,k}\cdot \epsilon_{a,b,c}^{\mathrm{inh}}\right)-\phi^{\mathrm{inh}}$$

(16) $$P_{x,y,\theta }\leftarrow P_{x,y,\theta }+\delta P_{x,y,\theta }^{\mathrm{exc}}+\delta P_{x,y,\theta }^{\mathrm{inh}}$$

where $\phi^{\mathrm{inh}}$ is an additional global inhibition constant used to suppress the energies of other pose cell clusters with relatively high energies.

The pose cell network takes two types of inputs:

1. Visual templates from the local view cell module;
2. Odometry estimation from the front-end LiDAR odometry algorithm.

For the visual template input, there are two kinds of operations depending on whether the visual template is new or a historical one. If it is a new visual template, then it will be associated with the centroid of the current energy packet in the pose cell network. If it is a historical one, then the corresponding energy will be injected into the pose cells previously associated with that visual template through the excitatory connection coefficient $\gamma^{\mathrm{exc}}$:

(17) $$\delta P_{x,y,\theta }=\alpha \cdot \sum_{j}\left(\gamma_{j,x,y,\theta }^{\mathrm{exc}}V_{j}\right)$$

where the constant coefficient α determines the degree of influence of visual information input on robot pose estimation. *P* and *V* represent the energy values of pose cells and local view cells, respectively.

For the odometry estimation input, we first perform the aforementioned local excitation and global inhibition steps, injecting and then removing a certain amount of energy around each active pose cell. Then, we normalize the energy of the whole pose cell network, maintaining its stability. Next, path integration is implemented using odometry information from the front end. Energy is transferred within the pose cells, facilitating the movement of the energy packet, the centroid of which in the current pose cell network is identified as the optimal estimate of the robot’s current pose within the network.

After these steps, the pose cell network outputs topological graph instructions and passes them to the experience map module. The topological graph instructions include:

1. Creating a new node (along with creating an edge from the previous node to the new node);
2. Creating an edge between two existing nodes;
3. Setting the current pose as an existing node.

#### 2.2.3. Experience Map

The experience map represents the map in the form of a graph, combining information from the pose cell network and local view cells to uniquely estimate the robot’s pose. The node in the experience map is defined as

(18) $$e_{i}=\left\{P_{i},V_{i},p_{i}\right\}$$

where $P_{i}$ and $V_{i}$ represent the energy values of the pose cells and local view cells, respectively, and $p_{i}$ represents the pose in the experience map space. And the edge in the experience map is defined as

(19) $$l_{ij}=\left\{\delta p_{ij},\delta t_{ij}\right\}$$

where $\delta p_{ij}$ and $\delta t_{ij}$ represent the relative differences in pose and time between two experience nodes $e_{i}$ and $e_{j}$.

The experience map is updated based on the topological graph instructions given by the pose cell network, which include creating nodes, creating edges, or setting nodes.

**Figure 7:**
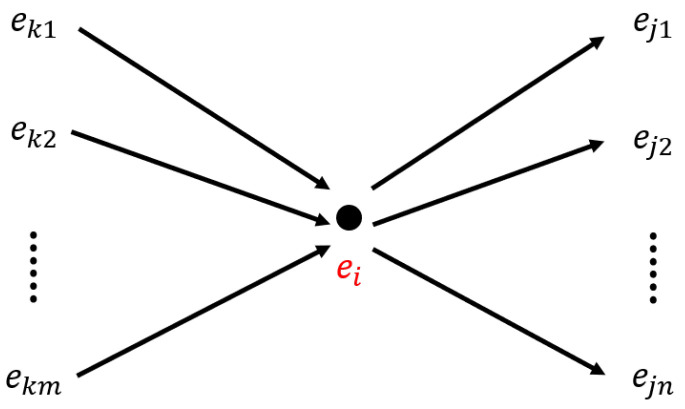
Connection relationships of the experience map. $e_{j1},e_{j2},\ldots ,e_{jn}$ are experience nodes that $e_{i}$ points to, and $e_{k1},e_{k2},\ldots ,e_{km}$ are experience nodes that point to $e_{i}$.

Additionally, a graph relaxation algorithm is applied to reduce the drift of the odometry estimation, where the pose $p_{i}$ is modified as

(20) $$\delta p_{i}=\xi \cdot \left(\sum_{j}\left(p_{j}-p_{i}-\delta p_{ij}\right)+\sum_{k}\left(p_{k}-p_{i}-\delta p_{ki}\right)\right)$$

(21) $$p_{i}\leftarrow p_{i}+\delta p_{i}$$

where ξ is the relaxation factor, $e_{j}$ represents any experience node that $e_{i}$ points to, and $e_{k}$ represents any experience node that points to $e_{i}$, whose connection relationships are illustrated in Figure 7.

## 3. Results

In this section, we validate the proposed LFVB-BioSLAM through the following real-world experiments using evaluation metrics that are designed to assess its localization and mapping performance.

### 3.1. Experimental Setup

**Figure 8:**
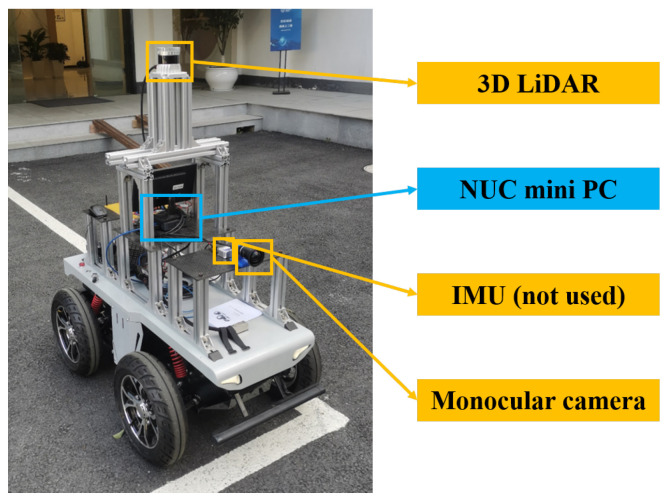
The ground mobile robot platform for data collection. The sensor system consists of a 3D LiDAR, an inertial measurement unit (IMU, not used in this implementation), and a monocular camera. The platform is equipped with an NUC mini PC running the necessary sensor drivers and collecting the datasets.

The ground mobile robot platform equipped with the sensor system is illustrated in Figure 8.

**Figure 9:**
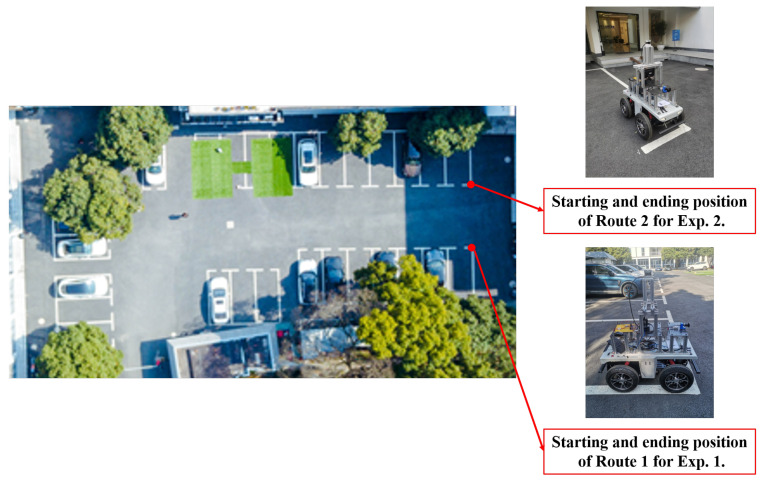
The bird’s-eye view of the experimental field with the starting and ending positions of the two routes corresponding to Exp. 1 and Exp. 2, respectively.

The experimental field was chosen as a courtyard with a length of approximately 50 m and a width of approximately 20 m. Two groups of experiments (denoted as Exp. 1 and Exp. 2) were conducted, in each of which the ground mobile robot was remotely operated along a corresponding predetermined route (denoted as Route 1 and Route 2, respectively) to collect data from the sensors. The bird’s-eye view of the experimental field along with the starting and ending positions of the two routes are shown in Figure 9.

### 3.2. Experimental Results

We adopt DLO (direct LiDAR odometry) [37], which is among the state-of-the-art (SOTA) algorithms in the field of LiDAR odometry and LiDAR SLAM, to obtain the relatively accurate robot odometry results using the 3D LiDAR point cloud data. The results of DLO serve as a benchmark against which the performance of the following three algorithms are evaluated:

1. RatSLAM [21], a classical bio-inspired visual SLAM, with a similar bio-inspired back-end processing mechanism to our LFVB-BioSLAM;
2. RF2O [32], a range flow-based horizontal planar laser odometry, with a similar processing mechanism to the front-end odometry estimation algorithm in our LFVB-BioSLAM;
3. LFVB-BioSLAM proposed by us, with a LiDAR-based front end and a vision-based bio-inspired back end.

**Figure 10:**
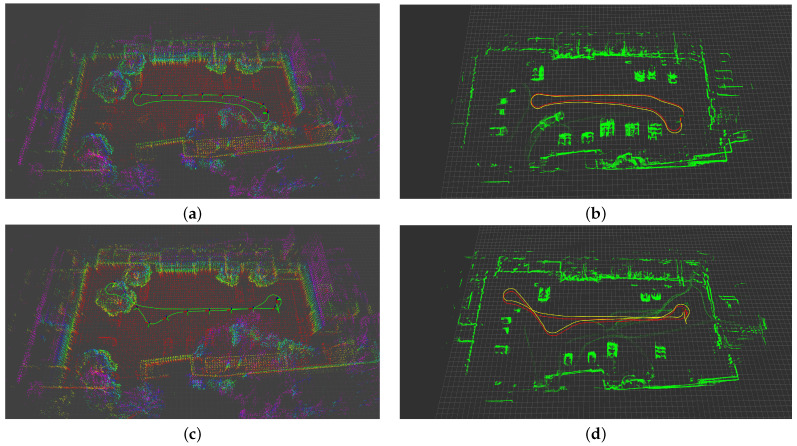
Results of robot poses and trajectories and environment maps generated by each algorithm. (**a**) Exp. 1, DLO. (**b**) Exp. 1, RF2O (in yellow) and LFVB-BioSLAM (in red). (**c**) Exp. 2, DLO. (**d**) Exp. 2, RF2O (in yellow) and LFVB-BioSLAM (in red).

**Figure 11:**
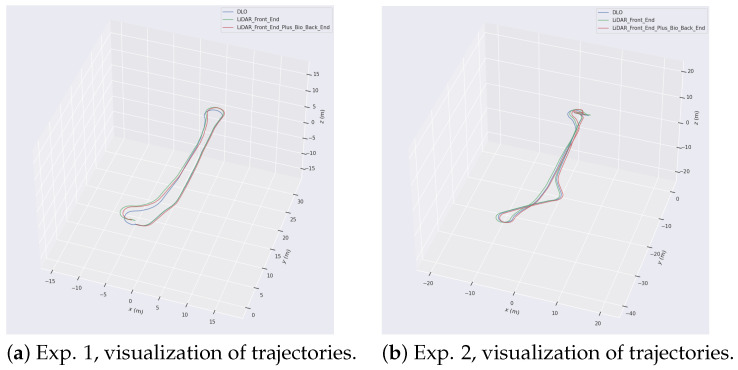
Visualization of robot trajectories generated by each algorithm in Exp. 1 and Exp. 2. The result of DLO, RF2O, and LFVB-BioSLAM are denoted as *DLO*, *LiDAR_Front_End*, and *LiDAR_Front_End_Plus_Bio_Back_End*, respectively.

The best parameter configurations for different algorithms are listed in Table 1. The poses and trajectories of the robot, as well as the constructed environment maps generated by these algorithms, are shown using RViz in Figure 10. Moreover, the open-source EVO [38] toolkit is used for the further visualization of robot trajectories obtained by each algorithm, as shown in Figure 11, providing an intuitive understanding of the accuracy performance of each algorithm.

It is worth mentioning that RatSLAM fails to produce reliable results in both Exp. 1 and Exp. 2 as it exhibits significant drift, resulting in localization failure. Therefore, the results obtained from the RatSLAM algorithm are not depicted in these figures.

**Figure 12:**
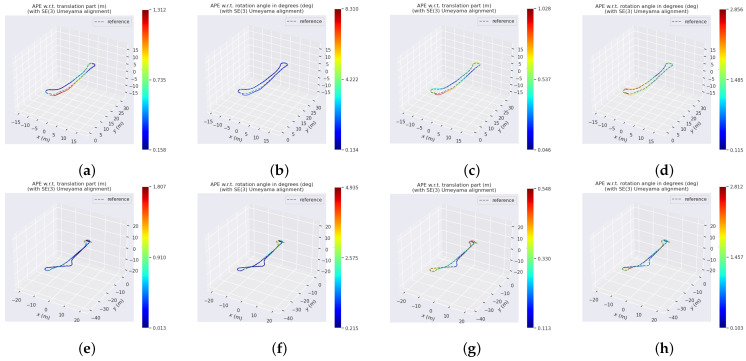
Visualization of the Translational and Rotational APEs of RF2O and LFVB-BioSLAM using the results of DLO as benchmark values for reference. (**a**) Exp. 1, Trans. APE of RF2O. (**b**) Exp. 1, Rot. APE of RF2O. (**c**) Exp. 1, Trans. APE of LFVB-BioSLAM. (**d**) Exp. 1, Rot. APE of LFVB-BioSLAM. (**e**) Exp. 2, Trans. APE of RF2O. (**f**) Exp. 2, Rot. APE of RF2O. (**g**) Exp. 2, Trans. APE of LFVB-BioSLAM. (**h**) Exp. 2, Rot. APE of LFVB-BioSLAM.

In order to quantitatively evaluate the accuracy performance of the aforementioned algorithms, the translational absolute pose error (Trans. APE) and rotational absolute pose error (Rot. APE) are selected as the evaluation metrics. The maximum error (max), mean error (mean), and root mean square error (RMSE) are calculated to compare the errors between the results of each algorithm and the benchmark values obtained from DLO. The comparison results of Translational APE and Rotational APE are illustrated in Table 2 and Table 3, respectively, which can also be visualized in Figure 12.

## 4. Discussion

In the experiments presented above, the performances of RatSLAM, RF2O, and the proposed LFVB-BioSLAM are compared against each other using DLO as the benchmark algorithm. Compared with the other two algorithms, the proposed LFVB-BioSLAM has significant advantages in terms of accuracy and robustness. The qualitative comparison results of computational complexity, accuracy, and robustness can be summarized in Table 4.

**Figure 13:**
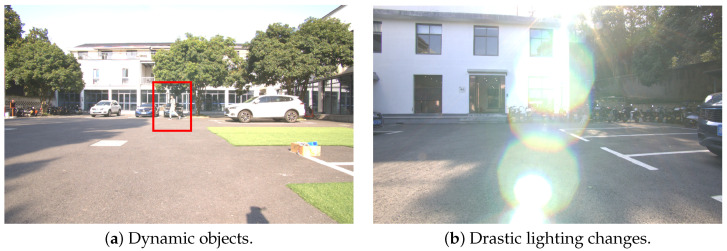
Scenarios in our experiments that are challenging for vision-based SLAM algorithms.

In both groups of experiments, RatSLAM failed due to significant localization drift, being incapable of performing localization and mapping tasks. The main reason is that RatSLAM uses a monocular camera as its single sensor, which fails to meet the practical navigation and localization requirements in certain experimental scenarios that are challenging for vision-based SLAM algorithms. For example, dynamic objects and drastic lighting changes in the environment, as shown in Figure 13, may lead to significant errors in the relatively simple front-end visual odometry and visual template matching steps of RatSLAM. These errors are difficult to correct in subsequent modules such as the pose cell network and the experience map, ultimately resulting in its failure with poor accuracy and robustness.

The proposed LFVB-BioSLAM, with the inclusion of the bio-inspired back end for loop closure detection and path integration, significantly outperforms RF2O in accuracy. The major function of the RF2O-like light-weight LiDAR odometry, which is the front end of LFVB-BioSLAM, is to provide fast and rough odometry estimation with low computational resource consumption, which will be further optimized by the bio-inspired back end. LFVB-BioSLAM also demonstrates good performance in the face of dynamic or partially degraded scenes with strong robustness.

## 5. Conclusions and Future Work

In this paper, we present LFVB-BioSLAM, a first-of-its-kind SLAM system consisting of a light-weight LiDAR-based front end and a bio-inspired vision-based back end, which, to the best of our knowledge, has not been previously proposed. The experimental results demonstrate that LFVB-BioSLAM outperforms RatSLAM, a vision-based bionic SLAM algorithm, and RF2O, a laser-based horizontal planar odometry algorithm, in terms of accuracy and robustness, validating the feasibility of the proposed bionic SLAM architecture.

In summary, the proposed bionic SLAM system, featuring an innovative framework, demonstrates commendable performance and practicality. This, to a considerable extent, facilitates the development of bionic SLAM research and lays a solid foundation for the future evolution of bionic SLAM systems tailored for fully autonomous robot navigation tasks.

For future work, more research on bionic SLAM remains to be done. On the one hand, from the perspective of bionics principles, bionic SLAM algorithms hold immense potential. By drawing inspiration from the latest theories about the animal navigation mechanisms in neural science, such as navigation cell collaboration mechanisms, it might be possible to construct a novel SLAM framework. The implementation of such a framework could potentially lead to breakthroughs in theoretical and technological advancements. On the other hand, neuromorphic hardware exhibits promising advantages, including asynchronous computing and event-based communication, along with ultra-low energy consumption, high parallelism, and efficiency. The application of neuromorphic hardware in bionic SLAM systems can provide assurance for achieving high real-time and energy-efficient performance, so the exploration of bionic SLAM algorithms based on neuromorphic hardware also carries significant research value.

## Figures and Tables

**Table 1:** Parameter configurations.

Algorithm	Parameter	Meaning	Value
RF2O & LFVB-BioSLAM	$l_{ctf}$	coarse-to-fine levels	5
	$i_{irls}$	IRLS iterations	5
	m	Gaussian mask	(0.0625, 0.25, 0.375, 0.25, and 0.0625)
RatSLAM & LFVB-BioSLAM	$y_{m}$	max *y* for image cropping	1000
	$th_{vt}$	visual template matching threshold	0.03
	$x_{vt}$	*x* size of visual template	60
	$y_{vt}$	*y* size of visual template	10
	$st_{vt}$	visual template matching step	1
	$n_{vt}$	visual template normalization factor	0.4
	$x_{pc}$	*x* size of pose cell	2
	$s_{pc}$	side length of (x,y) plane of pose cell network	100
	$l_{exp}$	experience map graph relaxation loops per iteration	20

**Table 2:** The comparison results of Translational APEs.

Experiment	Trajectory Length [m]	Algorithm	Trans. APE [m]		
			Max	Mean	RMSE
Exp. 1	74.76	RatSLAM *	/	/	/
		RF2O	1.3116	0.5484	0.6366
		LFVB-BioSLAM	**1.0280**	**0.5347**	**0.5835**
Exp. 2	101.76	RatSLAM *	/	/	/
		RF2O	1.8066	0.3913	0.4838
		LFVB-BioSLAM	**0.5480**	**0.2967**	**0.3136**

* Failure due to significant localization drift.

**Table 3:** The comparison results of Rotational APEs.

Experiment Number	Trajectory Length [m]	Algorithm	Rot. APE [${}^{\circ }$]		
			Max	Mean	RMSE
Exp. 1	74.76	RatSLAM *	/	/	/
		RF2O	8.3100	1.6831	1.8798
		LFVB-BioSLAM	**2.8556**	**1.6128**	**1.7079**
Exp. 2	101.76	RatSLAM *	/	/	/
		RF2O	4.9346	1.3323	1.6260
		LFVB-BioSLAM	**2.8120**	**1.0148**	**1.1193**

* Failure due to significant localization drift.

**Table 4:** The qualitative comparison results of each algorithm.

Algorithm	Computational Complexity	Accuracy	Robustness
RatSLAM	+	+	+
RF2O	++	++	++
LFVB-BioSLAM	+++	+++	+++

The greater the number of +, the higher the corresponding index.

## Data Availability

Not applicable.

## Abbreviations

The following abbreviations are used in this manuscript:

SLAM	Simultaneous localization and mapping
IMU	Inertial measurement unit
HDR	High dynamic range
SNN	Spiking neural network
STDP	Spike-timing-dependent plasticity
VPR	Visual place recognition
LO	LiDAR odometry
SAD	Sum of absolute differences
CAN	Continuous attractor network
SOTA	State-of-the-art
RMSE	Root mean square error
